# Modelling immune deterioration, immune recovery and state-specific duration of HIV-infected women with viral load adjustment: using parametric multistate model

**DOI:** 10.1186/s12889-020-08530-x

**Published:** 2020-03-30

**Authors:** Zelalem G. Dessie, Temesgen Zewotir, Henry Mwambi, Delia North

**Affiliations:** 1 School of Mathematics, Statistics and Computer Science, University of KwaZulu-Natal, Durban, South Africa; 2 College of Science, Bahir Dar University, Bahir Dar, Ethiopia

**Keywords:** Latent variables, Markov Chain, Orthogonal variable, Quality of life domain, Transition and waiting probabilities

## Abstract

**Background:**

CD4 cell and viral load count are highly correlated surrogate markers of human immunodeficiency virus (HIV) disease progression. In modelling the progression of HIV, previous studies mostly dealt with either CD4 cell counts or viral load alone. In this work, both biomarkers are in included one model, in order to study possible factors that affect the intensities of immune deterioration, immune recovery and state-specific duration of HIV-infected women.

**Methods:**

The data is from an ongoing prospective cohort study conducted among antiretroviral treatment (ART) naïve HIV-infected women in the province of KwaZulu-Natal, South Africa. Participants were enrolled in the acute HIV infection phase, then followed-up during chronic infection up to ART initiation. Full-parametric and semi-parametric Markov models were applied. Furthermore, the effect of the inclusion and exclusion viral load in the model was assessed.

**Results:**

Inclusion of a viral load component improves the efficiency of the model. The analysis results showed that patients who reported a stable sexual partner, having a higher educational level, higher physical health score and having a high mononuclear component score are more likely to spend more time in a good HIV state (particularly normal disease state). Patients with TB co-infection, with *anemia,* having a high liver abnormality score and patients who reported many sexual partners, had a significant increase in the intensities of immunological deterioration transitions. On the other hand, having high weight, higher education level, higher quality of life score, having high RBC parameters, high granulocyte component scores and high mononuclear component scores, significantly increased the intensities of immunological recovery transitions.

**Conclusion:**

Inclusion of both CD4 cell count based disease progression states and viral load, in the time-homogeneous Markov model, assisted in modeling the complete disease progression of HIV/AIDS. Higher quality of life (QoL) domain scores, good clinical characteristics, stable sexual partner and higher educational level were found to be predictive factors for transition and length of stay in sequential adversity of HIV/AIDS.

## Background

HIV infection is one of the leading causes of death from infectious diseases and remains a serious *global public health issue* [1, 2]. AIDS, the last progression stage of HIV infection, leads to severe damage of the body’s immune system [3]. The progression of HIV/AIDS is highly variable between populations and individuals and is determined by immunological, genetic, environmental and virological factors [4]. CD4 cell and viral load counts have remained the two strongest correlates and surrogate markers of HIV disease progression regularly used in the clinical setting to monitor the infection [5].

Although the main markers of HIV disease progression are both viral load and CD4 count, relatively few HIV/AIDS disease progression modelling studies include the longitudinal measurements of viral load biomarker along with clinical states of HIV/AIDS disease progression [6, 7]. This might be due to the unavailability of viral load data, particularly from middle and low-income countries, because of the higher costs of viral load testing [8]. Modelling of CD4 count progression, which takes into account the viral load biomarker, may better capturer the complete disease progression. Researchers further argue that modeling of disease progression of HIV/AIDS is important: to understand HIV pathogenesis and in the development of treatment strategies [9]; to refine the management of treatment-naive patients [10]; to determine at what threshold it is most clinically effective and less costly to begin ART [10]; to improve the empirical basis for epidemiological and prognostic models of the impact and cost-effectiveness of ART [11].

Modelling of disease progression of HIV/AIDS has been studied by several authors. Mangal [12] evaluated the effects of regional and age-specific differences on mortality and CD4^+^ cell progression of HIV/AIDS, using a hidden Markov model. Binquet et al. [13] estimated the impact of CD8 cell count, weight loss, drug use, gender, viral load and haemoglobin on the progression of HIV using multi-state Markov process. Oliveira et al. [14] studied the degrees of chronicity of HIV/AID using a multi-state process and went further to examine the impact of covariates; adherence, age and disease duration on CD4 cell count progression. Gillis et al. [15] further investigated the effects of age, gender, ethnicity, and people who had injected drugs, on the transitioning among the five viral load and CD4 cell counts based states. Recently, Shoko and Chikobvu [16] analyzed the effects of gender, TB co-infection, and age on transmission intensities, using the Markov model. Another study [17] in South Africa examined the effects of baseline viral load, gender, age and non-adherence to treatment on disease progression based on viral load followed by the death state using the Markov model. All these studies, however, did not model the length of stay in each state. In addition, although the factors related to disease progression of HIV are multiple and complex, no previous study directly examined the effects of several clinical variables (ie: white blood cell parameters, RBC parameters, blood chemistry parameters, and QOL domain scores) on both the length of stay and transitions of sequential events. This study thus gives a deeper insight on assessing the effect of several prognostic factors on both the transitions and length of stay of sequential adversity of HIV/AIDS.

In this study, full-parametric and semi-parametric multi-state Markov models are used to model the transitions and length of stay of sequential adverse events of HIV/AIDS. Multi-state Markov models are a powerful tool for studying chronic diseases and the factors associated with transitions between states of progression [14]. These models can accommodate competing risk factors, censored data, recurrent outcomes, multiple outcomes and frailty [18]. We classified the sequential adverse events by the degree of chronicity based on the CD4 counts (a marker for characterizing the *clinical stages)*, with progression classes defined by patients going through normal, mild, advanced and severe clinical stages [19]. More importantly, we presented full and semi-parametric multi-state Markov models on both the length of stay and transitions between sequential states, thus making this research different from previous studies. In addition to that, among the determinants of the progression of HIV/AIDS, both the CD4 cell counts and viral load counts are included in the same model. As discussed by Chikobvu and Shoko [17], the effects of multi-collinearity on the CD4 cell count transitions can be corrected using the principal component approach.

## Methods

### Data description

The data is from an ongoing prospective cohort study conducted by the Centre for the AIDS Program of Research in South Africa (CAPRISA) among ART naïve HIV-infected women. The original study, CAPRISA_002, which started in 2004, enrolled a cohort of HIV uninfected women whose age was greater than 18 years with the aim to describe immunologic, clinical and virologic characteristics of HIV-1 disease [20]. The study enrollment was conducted from August 2004 to December 2017. A participant who seroconverted during the HIV uninfected stage of CAPRISA_002 and other CAPRISA prevention and seroincidences trials (including the CAPRISA_004 trials), were enrolled into the Acute HIV Infection phase, and then followed-up during chronic infection and up to ART initiation. Participants were recruited at two sites in KwaZulu-Natal-South Africa, a rural site in Vulindlela and an urban site in the city of Durban. Women without well documented estimated date of HIV infection, and those who did not have at least two follow-up clinical attribute measurements were excluded from our analyses. Finally, 219 participants were included in the study. Further information about the above mentioned ongoing prospective HIV cohort study (CAPRISA_002), including women eligibility criteria and the enrollment procedures were reported in [20–22].

### Variables and measurements

CAPRISA initially enrolled HIV-negative (phase I) women into different study cohorts. The women who seroconverted were enrolled into the acute infection phase (i.e. phase II: weekly visits up to 3 months post-infection), early infection phase (i.e. phase III: monthly visits from 3 to 12 months), established infection phase (i.e. phase IV: quarterly visits for more than 12 months) and on ART phase (i.e. phase V). Samples for immunological, virological and clinical attributes (such as viral load, WBC parameters, RBC parameters, blood chemistry parameters, CD4 cell count, etc.) were measured at each visit [23]. These longitudinal immunologic, virologic and clinical measurements, were recorded for several followed-up visits. There was a total of 8760 follow-up visits recorded from 219 HIV infected women. Of these patients, 9.2% of them were co-infected with TB and all were black females, with a median age of 25 years (Interquartile range, IQR, 22–30). Over half (69.9%) reported having completed grades 11/12 of schooling. The median baseline CD4 count of the participants included in the analysis was 519.0 cells/mm3 (IQR 419–655.5 cells/mm3). The VL count of the participants ranged from 1.47 log_10_ copies/ml to 6.81 log_10_ copies/ml with the first quartile of 3.56 log_10_ copies/ml, a median of 4.23 log_10_ copies/ml and the third quartile of 4.79 log_10_ copies/ml.

**Fig. 1:**
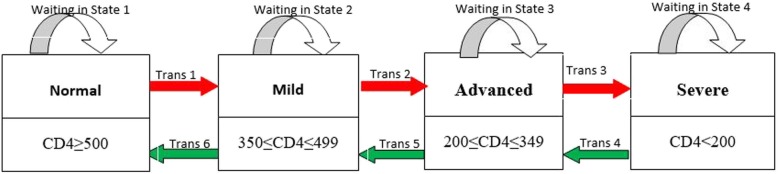
Graphical display of the hypothesized model

The main outcome variables in this current paper are immune deterioration, immune recovery and state-specific duration of stay of HIV-infected women. During the follow-up period, a patient could go through several states defined as normal, mild, advanced and severe disease states (See Fig. 1). The World Health Organization immunological classifications were used to assess the degree of severity of HIV infection of patients in the study. These HIV infection states are: no adverse events (normal) (CD4≥  500), mild (350≤CD4≤499), advanced (200≤CD4≤349) and severe (CD4 < 200) [19].

**Fig. 2:**
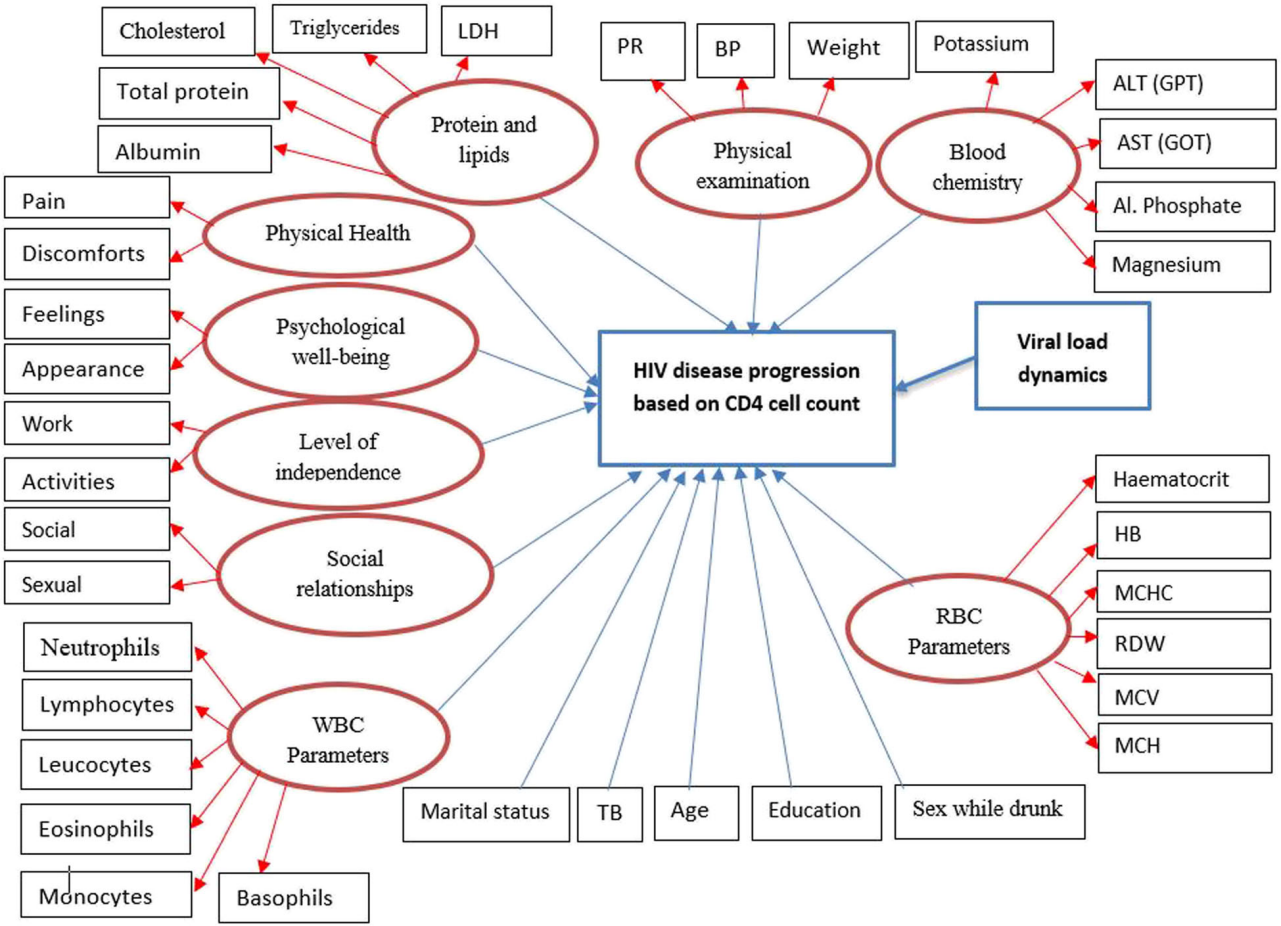
The four-state Diagram for HIV Progression of patients based on CD4 counts. Note: immunological recovery (green arrows), immunological deterioration (red arrows) and waiting time (black and white arrows)

The effect of numerous possible factors on the intensities of immune deterioration, immune recovery and state-specific duration of stay of HIV-infected women, were evaluated including, (1) demographic variables, (2) risk variables, (3) past opportunistic illness/infections and (4) clinical attributes and (5) health-related quality of life domain scores (HR-QoL). The WHO QoL instrument [24], was used to measure the HR-QoL of the participants. Therefore, the HR-QoL scales contain the following four domains score. The first domain is the physical health scores, that measure the impact of the disease on the activities of daily living, dependence on therapeutic substances, presence of pain, fatigue, lack of energy and initiative and perceived working capacity. The second is the psychological wellbeing score domain, that assesses the patient’s thoughts about body appearance, positive and negative feelings, self-esteem and personal beliefs, higher cognitive functions, spirituality, anxiety, suicide and depression. The third is the social relationships domain, which assesses personal relationships, social contacts, social support and sexual activity. The fourth domain is devoted to the level of independence and assesses areas such as mobility, activities of daily living, dependence on treatments and work capacity (See Fig. 2).

### Statistical method

#### Factor analysis

Since our data have a large number of clinical variables, we used exploratory factor analysis in order to group and minimize the number of variables. Accordingly from the 24 clinical variables in the study, we managed to group them in order to create 9 latent variables, defined as granulocytes components, mononuclear components, eosinophils component, RBC component, red blood cell indices, liver abnormality component, electrolyte component, lipid component and protein component. (See Table 1).

**Table 1:** Clinical parameters and corresponding factor loadings from the rotated factors

Clinical parameters	Principal Components	Variables	Rotated factor loadings	Commutative variations
White blood cell parameters	1. Mononuclear component	Lymphocytes	**0.838**	77%
		Basophils	**0.616**	
	2. Granulocytes component	Leucocyte	**0.925**	
		Neutrophils	**0.936**	
		Monocytes	**0.635**	
	3. Eosinophils component	Eosinophils	**0.947**	
Red blood cell parameters	4. Hb and haematocrit component	RBC counts	**0.946**	81%
		Hb	**0.886**	
		Haematocrit	**0.919**	
	5. RBC indices component	MCV	**0.953**	
		MCH	**0.825**	
		MCHC	**0.521**	
		RDW	**−0.592**	
Blood chemistry	6. Liver enzyme abnormality component	ALT(GPT)	**0.829**	72%
		AST (GOT)	**0.967**	
	7. Electrolyte component	Chloride	**0.455**	
		Sodium	**0.994**	
		Calcium	**0.213**	
Protein and lipids	8. Protein Comp	LDH	**−0.769**	75%
		Total protein	**0.670**	
	9. Lipid component	Cholesterol	**0.971**	
		LDL	**0.917**	
		Triglycerides	**0.360**	

#### Multi-state Markov modelling

Let a Markov chain process {*S*(*t*), *t* ∈ *T*}, *T* = [0, *τ*] for *τ* < ∞, that has finite space, denoted by *E* = {1, 2, 3, 4}, be a representation of the transition process, where for each patient, a multi-state process is observed. This Markov chain process has an initial probability, denoted by P(*S*(0) = *m*), *m* ∈ *E*, which evolves over time and with a history (*H*_*E*_), which contains the states previously visited, durations and times of transitions [25, 26]. The transition probability of the individual being in state j at time t, given that the individual was in state m at time z, is defined by

$$ {P}_{mj}\left(z,t\right)=P\left(S(t)=j|S(z)=m,{H}_E\right)\kern1.5em for\ m,j\in E\  and\ z,t\in T,z<t. $$

The corresponding transition intensity is defined by

$$ {h}_{mj}(t)=\underset{\delta t\to 0}{\lim}\frac{P\left(S\left(t+\delta t\right)=j|S(z)=m\right)}{\delta t}\kern1.5em for\ m\ne j.\kern12.75em $$

Where ${\sum}_{j\in E}{h}_{mj}(t)=0$ and ${h}_{mm}(t)=-{\sum}_{m\ne j}{h}_{mj}(t)$. It is worth noting that the transition probabilities depend only on the elapsed time, not on the times since the baseline of the study.

Besides modeling the ordinal transitional state of the CD4 progression, our object is to examine the effect of covariates or risk factors namely the education, marital status, age, sex under the influence of alcohol, TB-co infection status, anemia status, white blood cell parameters, RBC parameters, HR-QoL domain scores and weight on such transitions. We denote this set of covariates by **X.**

We employ both fully-parametric and semi-parametric multi-state models in our analysis. In both model types

$$ {h}_{mj}\left(t;\boldsymbol{X}\right)={h}_{mj}^0(t)\exp \left({\boldsymbol{\alpha}}_{mj}^{\prime }{\boldsymbol{X}}_{mj}\right)\kern18.25em $$

where. ${h}_{mj}^0(t)$ represents the baseline intensity from state *m* to state *j* and ***X***_*mj*_ representing a set of covariates, while ***α***_*mj*_ is the effect of the covariates on the hazard intensity *h*_*mj*_. The transition *m* to *j* is defined as immunological deterioration if *m* < *j*, as immunological recovery if *m* > *j;* as the probability of staying in the same diseasing state *if m* = *j*. In other words, the state labeling is 1 for normal, 2 for mild, 3 for advanced and 4 for severe states (See Fig. 1).

In the semi-parametric case, the log-linear effect of the covariates, ***α***_*mj*_ are estimated by the maximum partial likelihood and the corresponding baseline intensity, ${h}_{mj}^0(t)$, is left unspecified and estimated non-parametrically, similar to the Cox (1972) model.

In the fully-parametric cases, the baseline intensity, ${h}_{mj}^0(t)$, is given by a fully-parametric function of time, so that each transition-specific model is a standard parametric survival model. The log-linear effect of the covariates, ***α***_*mj*_, are estimated by full maximum likelihood and standard errors are obtained by standard asymptotic theory. In the current study, we used different parametric and semi-parametric models including the Exponential distribution and Weibull distribution.

### Principal component analysis

Principal component analysis (PCA) is a statistical procedure that uses an orthogonal transformation to convert a set of observations, of possibly correlated variables, into a set of values of linearly uncorrelated variables. In this paper, a principal component variable is created by fitting a regression model of log_10_-transformed viral load values (*y*_***i***_) on CD4 cell counts (*x*_***i***_) to improve the efficiency of the model above. In order to create two new uncorrelated components, as explained by Chikobvu and Shoko [17], we carried out regression analysis to estimate the intercept (β_0_) and slope (β_1_) parameters in the model: *y*_***i***_ = *β*_0_ + *β*_1_*x*_***i***_***+****ε*_*i*_. We then define a viral load orthogonal variable (VLO)= *ε*_*i*_**=** *y*_*i*_ − (*β*_0_ + *β*_1_*x*_*i*_)**.**

VLO in the model explains the component of disease progression of HIV/AIDS that cannot be explained by the CD4 counts alone. In order to deal with multicollinearity of CD4 cell count and viral load count, the orthogonal viral load component was used. The residual from the fitted model was included with the original HIV/AIDS data to form the new viral load component.

### Model diagnostics

The estimates of full and semi-parametric multi-state models were compared with non-parametric estimates to assess model fit (as discussed by Ieva et al. [27] and Titman and Sharples [28]). Besides selecting the best fit model for our data, the effect of CD4 count with viral load adjustment on the HIV/AIDS disease progression was also analyzed. This was done by fitting a multi-state model for the effects of possible factors (education, marital status, TB-co infection status, anemic status, age, sex under the influence of alcohol, white blood cell parameters, RBC parameters, HR-QoL domain scores, and weight) on disease progression of HIV/AIDS, based on CD4 count. Notably, we excluded the orthogonal viral load component in the first model. In the second model, viral load component was included in the modified first model. A comparison of these two models was based on their likelihood ratio test (LRT) and Akaike information criteria (AIC).

All of the analyses were implemented using R-3.6.2 (https://cran.r-project.org/bin/windows/base/) flexsurv, survival and mstate packages in order to fit fully-parametric Markov, Semi-parametric Markov and to estimate the nonparametric Aalen-Johansen model, respectively. For visual graphical presentation of the estimates proc. SGPLOT in SAS 9.4 (https://www.sas.com/en_us/software/sas9.html) was used.

## Results

### Estimated transition probability and length of stay

**Fig. 3:**
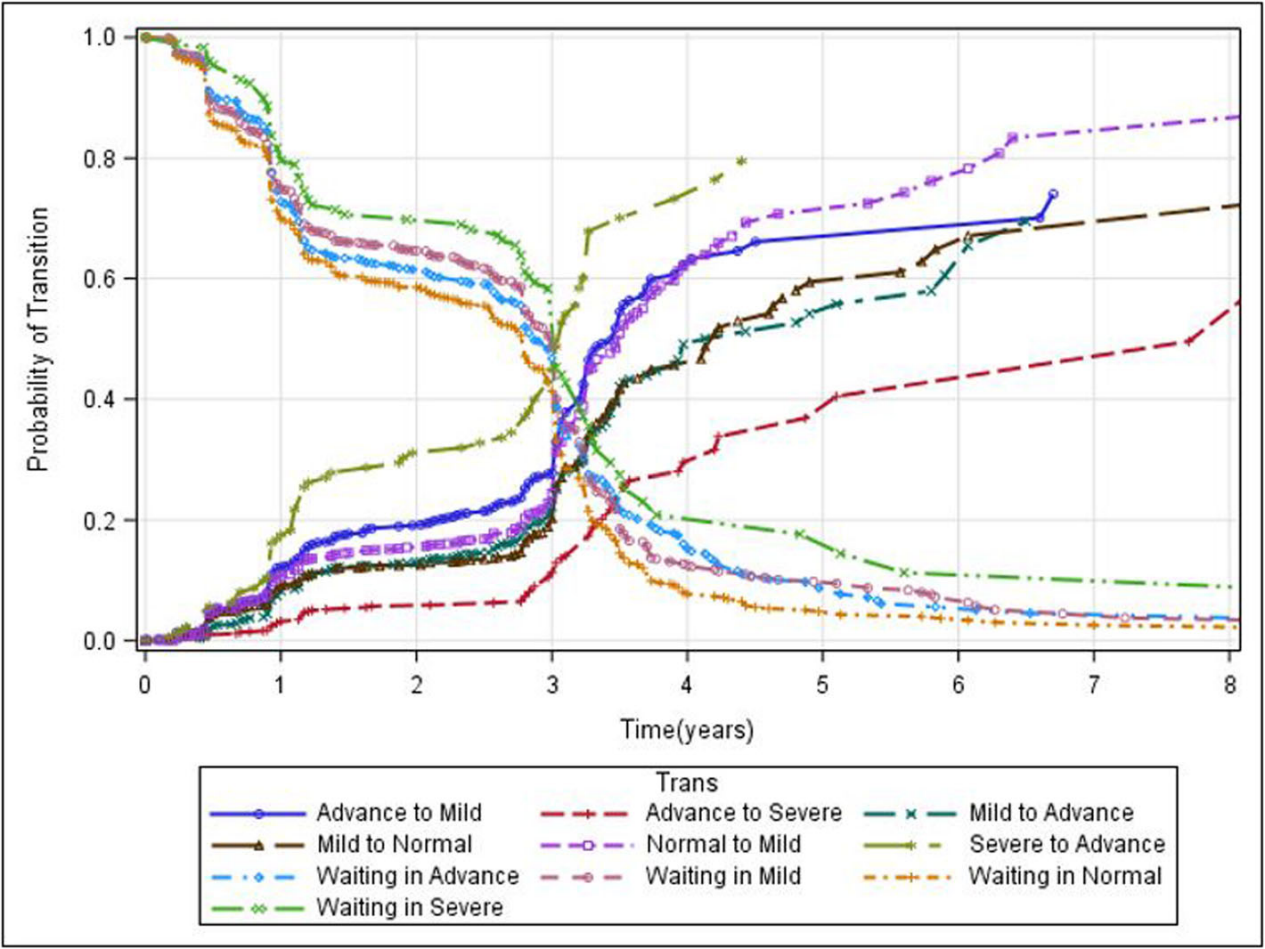
Estimated probability of transition and probability of being in each disease state of patients through the follow-up time

The plot in Fig. 3 displays the non-parametric Aalen-Johansen estimator of the transition and waiting probabilities. As expected, the probability of transition from severe to advanced states of the diseases did not increase much throughout follow-up, while the transition probability from advanced to severe disease states of the patients increased with increasing years since enrollment. The plots further clearly demonstrate that the overall probability of staying in the same diseasing state had decreased throughout the follow-up periods. Patients with lower CD4 count (particularly those in the severe disease state) had a higher probability of staying in the same state throughout follow-up periods, compared to those with higher CD4 cells counts. (See Fig. 3).

### Model assessment

**Fig. 4:**
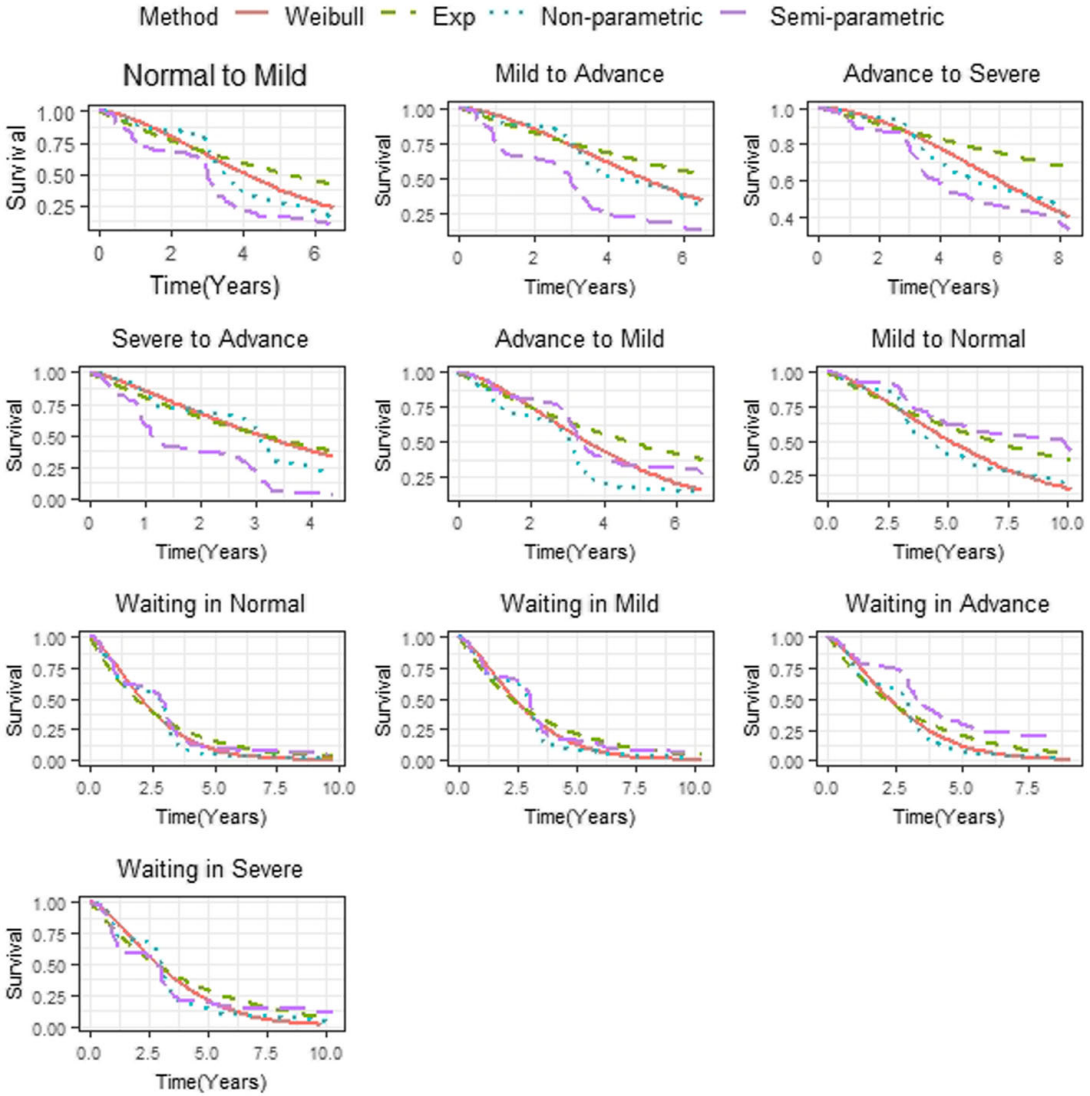
Goodness-of-fit the proposed model. Note: semi-parametric (purple), Weibull distribution (red) and Exponential distribution (green) transition and waiting probabilities estimates overlaid on non-parametric (blue) survival functions of time to initiation of ART, from the starting state

We applied three Markov multi-state models, including Exponential, Weibull and the Semi-parametric multi-state model. The overall goodness of fit of the models is presented in Fig. 4, which shows the estimates of full and semi-parametric models to be overlaid on the nonparametric Aalen-Johansen estimates. As seen from this plot, we noted that the Weibull model accounted for the decrease in the hazard of waiting and transition probability better compared to the Exponential and the Sem-parametric models. The model selection criteria in Table 2 also confirmed this finding.

**Table 2:** Model selection criteria for each semi and full-parametric model

Criterion	Weibull Multistate Markov Model	Exponential Multistate Markov Model	Semi-parametric Multistate Markov Model
−2 LOG L	**19148.12**	19,789.32	62,023.91
AIC	**19408.12**	20,147.32	62,589.36

Key: *AIC* Akaike information criteria, -*2 LOG L* -2Log-likelihood

The AIC and LRT from Table 3 showed that the model defined by CD4 cell counts states with the viral load orthogonal adjustment gives the best fit to the data (ie. improve the efficiency of the model). (See Table 3).

**Table 3:** Assessment of the fitted model with and without Viral Load Counts Component

Criterion	Without Orthogonal Viral Load Counts Covariate	With Orthogonal Viral Load Counts Covariate
-2 LOG L	19148.12	**18388.20**
AIC	19408.12	**18768.20**

Key: *AIC* Akaike information criteria, *2 LOG L* -2Log-likelihood

### Predictors of immune deterioration and immune recovery

The results in Table 4 show that after adjusting for other covariates in the model, an increase in Hb and haematocrit component increases the intensities of transition from mild to normal disease state (aHR = 1.12; 95% CI: 1.01–1.25). Similarly, after adjusting other covariates, an increase in RBC indices score increases the intensities of transition from mild to normal disease state (aHR = 1.16; 95% CI: 1.04–1.30). The result further showed that patients with high mononuclear component scores, significantly reduced immune deterioration from normal to mild (aHR = 0.77; 95% CI: 0.67–0.88), mild to advanced (aHR = 0.63; 95% CI: 0.55–0.73) and advanced to severe (aHR = 0.64; 95% CI: 0.50–0.83) disease state. Moreover, an increase in granulocytes component scores reduces the intensities of immune deterioration transitions.

**Table 4:** Parameter effects (with 95% CI) of Socio-demographics variables, risk variables, HR-QoL domain scores and clinical measurements on the transition intensities for the CD4 based Weibull multistate Markov model

	Tran1: Normal to Mild	Tran 2: Mild to Advanced	Tran 3: Advanced to Severe	Trans 4: Severe to Advanced	Trans 5: Advanced to Mild	Trans 6: Mild to Normal
	Exp(β) (95% CI)	Exp(β) (95% CI)	Exp(β) (95% CI)	Exp(β) (95% CI)	Exp(β) (95% CI)	Exp(β) (95% CI)
Orthogonal Viral load	1.62 (1.34, 1.91)***	1.67 (1.39, 2.01)***	2.10 (1.45, 3.04)**	1.10 (0.78, 1.54)	0.90 (0.77, 1.06)	0.82 (0.70, 0.97)***
TB: Yes	2.08 (1.02, 4.71)*	1.52 (0.32, 7.29)	1.86 (1.05, 4.61)*	0.84 (0.59, 5.72)	1.84 (0.41, 8.28)	0.79 (0.47, 1.32)
Age_Cat: 18–20 years	1.18 (0.72, 1.93)	1.09 (0.68, 1.77)	1.05 (0.53, 2.09)	0.39 (0.16, 2.04)	1.31 (0.88, 1.97)	1.07 (0.68, 1.67)
Age_Cat: 21–39 years	0.96 (0.56, 2.66)	1.23 (0.64, 2.54)	1.70 (0.06, 5.93)	0.41 (0.16, 1.00)	1.76 (0.71, 4.38)	1.88 (0.95, 3.73)
Anemia: Yes	1.23 (0.99, 1.52)	1.31 (1.04, 1.66)**	1.55 (0.92, 2.63)	0.59 (0.36, 0.98)**	0.94 (0.72, 1.24)	0.79 (0.61, 1.03)
Education: Grade 9–10	0.34 (0.18, 0.63)**	0.72 (0.40, 1.31)**	1.07 (0.52, 2.20)	1.11 (0.43, 2.91)	1.08 (0.59, 1.96)	1.20 (0.68, 2.11)
Education: Grade > 11	0.44 (0.25, 0.77)**	0.51 (0.29, 0.87)*	0.41 (0.23, 0.75)*	1.41 (1.57, 3.49)	0.91 (0.54, 1.52)	1.41 (0.88, 2.27)
Marital Status: Married/stable sexual partners	0.64 (0.32, 1.84)	0.60 (0.38, 1.06)	0.81 (0.34, 1.91)	1.31 (1.01, 4.92)*	1.10 (0.53, 1.97)	1.01 (0.61, 1.44)
Marital Status: Single/no sexual partners	0.58 (0.25, 3.88)	0.64 (0.26, 1.60)	0.63 (0.17, 2.29)	1.27 (1.01, 2.70)*	1.42 (0.37, 2.72)	1.08 (0.42, 2.42)
Sex while Drunk: yes	1.10 (0.72, 1.68)	0.84 (0.42, 1.70)	0.70 (0.29, 1.69)	1.46 (0.71, 2.99)	0.78 (0.44, 1.38)	1.05 (0.57, 1.94)
RBC indices component	0.98 (0.87, 1.10)	0.83 (0.75, 1.91)	0.93 (0.75, 1.15)	0.88 (0.73, 1.06)	0.92 (0.80, 1.05)	1.16 (1.04, 1.30)***
Hb and Haematocrit component	0.96 (0.86, 1.08)	1.00 (0.89, 1.13)	0.96 (0.78, 1.18)	1.16 (0.97, 1.38)	1.07 (0.94, 1.21)	1.12 (1.01, 1.25)**
Granulocytes component	0.91 (0.81, 1.03)	0.85 (0.76, 0.96)**	0.96 (0.78, 0.98)**	1.10 (0.88, 1.37)	1.18 (1.04, 1.34)*	1.14 (1.02, 1.28)**
Mononuclear component	0.77 (0.67, 0.88)**	0.63 (0.55, 0.73)***	0.64 (0.50, 0.83)**	0.95 (0.73, 1.23)	1.26 (1.13, 1.41)*	1.42 (1.26, 1.60)***
Liver enzymes abnormality component	0.94 (0.85, 1.05)	0.98 (0.88, 1.09)	1.05 (0.92, 1.21)	1.19 (0.90, 1.58)	0.86 (0.76, 0.98)*	1.06 (0.94, 1.20)
Weight	0.98 (0.97, 0.99)**	0.98 (0.97, 0.99)**	1.01 (0.99, 1.02)	1.02 (0.99, 1.04)	1.00 (0.99, 1.01)	1.00 (0.99, 1.01)
Psychological well-being scores	0.83 (0.76, 0.91)**	0.91 (0.82, 0.99)*	0.91 (0.78, 1.07)	0.85 (0.68, 1.05)	0.89 (0.82, 1.96)	0.87 (0.80, 1.95)
Physical health scores	1.21 (0.99, 1.33)	1.05 (0.94, 1.17)	1.03 (0.92, 1.17)	1.25 (1.01, 1.58)**	1.02 (0.96, 1.08)	1.17 (1.07, 1.29)**
Independence Score	0.87 (0.83, 0.91)*	0.96 (0.92, 1.00)	0.94 (0.88, 0.99)*	1.00 (0.90, 1.10)	1.01 (0.96, 1.05)	1.04 (0.99, 1.09)
Social relationship	0.96 (0.92, 0.99)*	0.97 (0.93, 1.02)	0.94 (0.86, 1.03)	0.90 (0.82, 1.00)	0.99 (0.95, 1.04)	0.92 (0.87, 0.97)

Key:- Statistical significance: (*)*P* < 0.05; (**)*P* < 0.01; (***)*P* < 0.001; reference category: Age [> 40]; education [Grade ≤ 8]; marital status [Many sexual partners]; TB [No]; Anemia [No]

After adjusting for other covariates in the model, an increase in liver abnormality score reduces the intensities of transition from advanced to mild disease state (aHR = 0.86; 95% CI: 0.76–0.98). Anemic patients had also significantly increased the intensity of immune deterioration from mild to advanced disease state (aHR = 1.31; 95% CI: 1.04–1.66) as compared to non-anemic patients. The result further showed that having TB co-infection significantly accelerates the transition time from normal to mild (aHR = 2.08; 95% CI: 1.02–4.71) and advance to severe (aHR = 1.86; 95% CI: 1.05–4.61) disease stages, compared to those without TB co-infection. (See Table 4).

With regard to HR-QoL variables, patients with high physical health scores significantly increased the intensities of immunological recovery from severe to advanced (aHR = 1.25; 95%CI: 1.01–1.58) and mild to normal disease state (aHR = 1.17; 95% CI: 1.07–1.29). Similarly, an increase in level of independence score reduces the intensities of transition from normal to mild (aHR = 0.87; 95% CI: 0.83–0.91) and advanced to severe disease state (aHR = 0.94; 95% CI: 0.88–0.99). Furthermore, having high psychological well-being scores, is significantly associated with reduced intensities of transitions from normal to mild (aHR = 0.83; 95% CI: 0.76–0.91) and mild to advanced (aHR = 0.91; 95% CI: 0.82–0.99) disease state (See Table 4).

With regard to socio-demographic variables, patients with higher educational levels (grade > 11) had statistically significantly reduced intensities of transitions from normal to mild (aHR = 0.44; 95% CI: 0.25–0.77), mild to advanced (aHR = 0.51; 95% CI: 0.29–0.87) and advanced to severe (aHR = 0.41; 95% CI: 0.23–0.73) disease state, as compared to those with low level of education (grade < 8). Patients who reported stable sexual partners (aHR = 1.31; 95% CI: 1.01–4.92) and no sexual partner (aHR = 1.27; 95% CI: 1.01–2.70), had significantly increased immune recovery, from severe to advanced disease state, as compared to those who reported many sexual partners. Furthermore, as the weight of women in the study increased, the intensities of immune deterioration from normal to mild and mild to advanced disease state, decreased (See Table 4).

### Predictors of state-specific duration of stay of HIV patients

After adjusting for education, marital status, TB-co infection, anemic status, age, sex while drunk, granulocytes component, RBC indices, HR-QoL domain scores, and weight, an increase in mononuclear component score increases the likelihood that a patient stayed in normal disease state (aHR = 1.27; 95% CI: 1.18–1.38) and mild disease state (aHR = 1.12; 95% CI: 1.04–1.21), (Table 5). The result further showed that patients with higher physical health scores were more likely to stay longer in normal (aHR = 1.07; 95% CI: 1.01–1.14) and mild disease state (aHR = 1.12; 95% CI: 1.05–1.21), but significantly reduced the waiting time in a severe disease state (aHR = 0.85; 95% CI: 0.73–0.99). Similarly, we noted that those having high psychological well-being scores and high social relationship scores were less likely to stay longer in severe disease state. (See Table 5).

**Table 5:** Parameter effects (with 95% CI) of Socio-demographics variables, risk variables, HR-QoL domain scores and clinical measurements on length of stay (waiting time) for the CD4 based Weibull multistate Markov model

Variables	Waiting in Normal	Waiting in Mild	Waiting in Advanced	Waiting in Severe
	Exp(β) (95% CI)	Exp(β) (95% CI)	Exp(β) (95% CI)	Exp(β) (95% CI)
Orthogonal Viral load	0.90 (0.81, 0.99)***	1.42 (0.60, 3.33)	1.27 (1.11, 1.45)**	1.28 (1.15, 1.43)**
TB: Yes	1.18 (0.34, 4.19)	0.99 (0.76, 1.29)	1.08 (0.75, 1.56)	1.44 (0.63, 3.30)
Age_Cat: 18–20 years	0.56 (0.39, 1.80)	1.11 (0.77, 1.17)	1.16 (0.91, 1.48)	0.22 (0.11, 1.45)
Age_Cat: 21–39 years	2.31 (0.07, 4.96)	1.05 (0.78, 1.47)	0.74 (0.54, 1.02)	0.02 (0.01, 1.03)
Anemia: Yes	1.12 (0.97, 1.30)	1.05 (0.91, 1.21)	1.08 (0.89, 1.30)	0.87 (0.51, 1.49)
Education: Grade 9–10	3.56 (1.34, 9.48)*	1.07 (0.79, 1.45)	1.33 (0.92, 1.94)	0.94 (0.61, 1.45)
Education: Grade > 11	4.80 (2.02, 11.40)*	0.87 (0.67, 1.14)	1.23 (0.92, 1.65)	0.91 (0.58, 1.43)
Marital Status: Married/stable sexual partners	1.51 (1.01, 2.25)*	1.27 (1.01, 2.05)*	0.98 (0.56, 1.73)	1.18 (0.34, 3.91)
Marital Status: Single/no sexual partners	1.71 (1.14, 2.78)*	1.26 (0.98, 1.63)	0.82 (0.62, 1.08)	2.60 (0.17, 16.19)
Sex while Drunk: yes	1.08 (0.78, 1.50)	0.95 (0.71, 1.26)	1.05 (0.77, 1.44)	1.41 (0.88, 2.28)
RBC indices component	1.05 (0.99, 1.12)	0.99 (0.92, 1.06)	0.94 (0.86, 1.03)	1.17 (0.99, 1.39)
Hb and Haematocrit component	1.11 (1.04, 1.19)	1.00 (0.93, 1.07)	0.94 (0.86, 1.02)	1.26 (0.99, 1.61)
Granulocytes component	0.98 (0.93, 1.03)	0.93 (0.88, 1.99)	0.99 (0.92, 1.07)	0.88 (0.66, 1.18)
Mononuclear component	1.27 (1.18, 1.38)**	1.12 (1.04, 1.21)**	1.07 (0.99, 1.15)	0.86 (0.64, 1.16)
Liver enzymes abnormality component	1.01 (0.95, 1.06)	0.99 (0.93, 1.05)	1.02 (0.95, 1.08)	0.87 (0.65, 1.18)
Weight	1.00 (0.99, 1.00)	1.00 (0.99, 1.00)	1.00 (0.99, 1.00)	1.01 (0.98, 1.04)
Psychological well-being scores	0.89 (0.83, 1.00)	0.96 (0.79, 1.17)	0.88 (0.83, 0.94)**	0.87 (0.81, 0.93)*
Physical health scores	1.07 (1.01, 1.14)**	1.12 (1.05, 1.21)*	1.06 (0.99, 1.12)	0.85 (0.73, 0.99)**
Independence scores	1.00 (0.97, 1.03)	1.00 (0.97, 1.03)	0.98 (0.96, 1.01)	0.95 (0.88, 1.03)
Social relationship scores	0.97 (0.93, 1.00)	0.95 (0.92, 1.00)	0.97 (0.93, 1.00)	0.96 (0.87, 0.99)*

Key:- Statistical significance: (*)P < 0.05; (**)P < 0.01; (***)P < 0.001; reference category: Age [> 40]; education [grade ≤ 8]; marital status [Many sexual partners]; TB [No]; Anemia [No]

After adjusting for education, marital status, TB-co infection, anemic status, age, sex while drunk, granulocytes component, RBC indices, HR-QoL domain scores, and weight, patients who reported stable sexual partnership (aHR = 1.51; 95% CI: 1.01–2.25) and no sexual partner (aHR = 1.71; 95% CI: 1.14–2.78) were more likely to stay longer in a normal disease state as compared to those who reported many sexual partners. Furthermore, patients with higher educational levels (grade > 11) were more likely to stay longer in the normal disease state (aHR = 4.80; 95% CI: 2.02–11.40), as compared to those with lower education (< 8 grade). (See Table 5).

## Discussion

We have presented both full-parametric and semi-parametric multi-state models, to model the transition intensity and length of stay of sequential adverse events of HIV/AIDS. We also improved the selected model, the Weibull multi-state model, by including an orthogonal viral load component, derived from PCA. The new orthogonal viral load component helped to explain the determinants of transitions and length of stay in sequential adverse events, which could not be explained by the CD4 count alone. This further improved the efficiency and predictive accuracy of the model. Results from the Akaike information criteria and likelihood ratio test showed that the model defined by CD4 cell counts states with the viral load orthogonal component, fitted significantly better than the model with the exclusion of orthogonal viral load components, as it was done by [17, 29].

Among the different hematological parameters for HIV infected patients, as expected, a latent variable related to basophils counts and total lymphocytes was significantly associated with the intensities of immunological deterioration transitions, and this confirms that HIV infection targets T-cells. Many studies also suggested that total lymphocytes can adequately serve as a surrogate biomarker for predicting CD4 count progression [30–32]. The latent variable related to RBC indices was significantly associated with CD4 count progression, which is in agreement with findings reported in previous studies [33–35]. The RBC indices result also shows that it could be used as a reliable marker of the prognosis in HIV- patient and that a therapeutic approach is imperative for a patient with anemia. Thus, hematological parameters such as basophils counts, total lymphocytes and red blood cell indices (i.e. MCV and MCH) could thus help health workers identify patients with poor immunological and clinical responses in the absence of CD4 count. Furthermore, the latent variable related to alanine aminotransferase and aspartate aminotransferase was significantly associated with the intensities of immunological deterioration transitions, and this confirms that the infection is the underlying cause of the increased activities of liver enzymes [36, 37]. A study in Ethiopia also reported similar findings [38], showing that lower CD4 count level (CD4 < 200 cells/mm3) was associated with elevated liver enzymes. Thus, there is a need to monitor alanine aminotransferase and aspartate aminotransferase levels before initiation of ART mainly in high-risk patients to reduce side effect concerns.

We found that hematological abnormalities such as anemia had a significantly accelerated immune deterioration effect. This may be attributed to the fact that anemia is commonly due to the under-production of erythrocytes by the bone marrow cells [39]. The bone marrow cells are also responsible for the production of CD4 cell counts through the myeloid cells [40]. Poor production of myeloid cells can, therefore, result in decreased production of both erythrocytes and CD4 cells. A study done in Ethiopia [41] reported similar findings that having low CD4+ T cells count level (<200cells/mm3) and being HAART-naïve, were significantly associated with anemia. Other researchers [42–44] further reported that more severe levels of anemia are found among HIV positive patients presenting with low CD4 count level.

We observed that TB co-infection accelerated the deterioration of immunological functions, a finding that is in accordance with the literature [45–48], where TB co-infection has a negative impact on the immune response to HIV, accelerating the progression from HIV infection to AIDS. Consequently, caution is needed for risk assessment measures to monitor and screen patient’s pre-ART initiation in African clinical settings, to curtail potential risks associated with an increased probability of accelerating the deterioration of immunological functions. Furthermore, in agreement with the world health organization treatment guidelines [49], which recommends ART in all PLHIV regardless of CD4 cell count, early identification of patients with poor clinical characteristics and initiation of treatment will improve programmatic success and treatment prognosis.

HIV patients who reported many sexual partners had significantly increased intensities of immune deterioration transitions. Moreover, patients who reported stable or no sexual partners were more likely to spend a long time in a normal disease state, as compared to those who reported many sexual partners. Indeed it has been reported [50] that patients with higher sexual risk-taking behaviors (such as many sexual partners) were significantly linked to depression, physically and psychologically impairment among patients living with HIV. Chronic depression and low quality of life scores may be associated with increased probability of experiencing immunological deterioration [51, 52], showing that at least part of the effect of many sexual partners on incomplete immunological recovery, is mediated through QoL and depression.

Patients with higher educational levels had significantly reduced rates of immune deterioration. Other researchers [53, 54] also found that higher education promotes a better rate of change of immune recovery, possibly due to better knowledge about their treatment and disease, access to health services, or functional status. We have also observed that patients having higher educational levels were significantly associated with longer time spent in normal disease states. This might be attributed to patients having higher educational levels were significantly associated with good sanitation and hygiene practices [55]. Good sanitation and hygiene practices decrease the risk of diarrhea, which can increase CD4 count [56]. This shows the effect of educational level on the time spent in healthier states (particularly the normal disease state), is mediated through good sanitation and hygiene practices. Another possibility may be that higher educational level often provides financial benefits and thus better access to nutritious food. This may be attributed to the fact that nutritious food significantly increases the time spent in healthier states.

Having high body weight significantly reduces the intensities of immunological deterioration transitions. This finding is consistent with higher weight was significantly related to higher CD4 cell count levels [57, 58]. Recent studies have also shown that patients with higher BMI at pre-HAART possess higher CD4 cell count and CD4/CD8 ratio compared to normal or underweight adults [59, 60].

A significant positive relationship between QoL domain scores and intensities of recovery of HIV infected patients is noted in our study. Previous studies [61–63] had reported a significant positive relationship between CD4 cell count recovery and HR-QoL scores of HIV infected patients. In contrast, studies [64, 65] from sub-Saharan Africa showed that there was no significant relationship between CD4 count level and HR-QoL scores. Possible explanations for this controversial finding might be that our data was from a cohort of acutely infected patients and followed up repeatedly over an extended ART-free period. We have also observed that patients having higher physical health scores were significantly associated with longer time spent in normal disease states. This may explain the observed positive progression in the physical health domain score and the CD4 cell counts. Furthermore, having high mononuclear component scores were significantly associated with longer time spent in normal disease states.

This study has some limitations, including missing data, which are expected for a study conducted on data collected from patients’ files with many variables and a long term follow-up period. Moreover, some clinical covariates that influence the intensities of immune deterioration and immune recovery, may not have been included, for example, CD4/CD8 ratio and hepatitis status. Furthermore, the study findings were limited to adult females.

## Conclusions

From a clinical perspective, the study has revealed that having higher QoL domain scores, good clinical characteristics, stable sexual partners and higher educational levels, were found to be the significant predictive factors for intensities of immune deterioration, immune recovery and state-specific length of stay of HIV-infected women. Furthermore, we found that the identification of factors, such as QoL measurement items, clinical attributes, marital status, and educational status, associated with the current state of the patient, were important contributing factors to extend the survival of the patients and could potentially guide clinical interventions.

From a methodological perspective, by treating the collinearity of the viral load and CD4 cell counts using orthogonal; transformation, the Weibull multi-state Markov model gives a better fit and insight than the semi-parametric methods. Moreover, our study will help researchers to uncover the critical areas of correcting and dealing with multicollinearity when including both CD4 cell and viral load count in parametric multi-state modelling of HIV/AIDS, that many researchers have not been able to explore. Other more robust parametric distributions other than the Weibull are also a possible area of further research.

## Data Availability

The dataset used and analyzed during the current study is available from the corresponding author on reasonable request.

## Abbreviations

*AIDS*: Acquired Immune Deficiency Syndrome
HB: Hemoglobin
V B12: Vitamin B12
BP: Blood pressure
LDH: Lactate dehydrogenase
PR: Pulse rate
RBC: Red blood cells
HR-QoL: Health-related quality of life
CAPRISA: Center of the AIDS Programme of Research in South Africa
OI: *Opportunistic infections*
*ARV*: Antiretroviral (drug)
ART: Antiretroviral therapy
HIV: Human immunodeficiency virus
MCHC: Mean corpuscular haemoglobin concentration
MCV: Mean corpuscular volume
MCH: Mean corpuscular haemoglobin
RDW: Red cell distribution width
ALT: Alanine aminotransferase
AST: Aspartate aminotransferase
